# Changes in dairy cows’ behaviour, health, and production after transition from tied to loose housing

**DOI:** 10.1186/s13028-023-00690-1

**Published:** 2023-06-30

**Authors:** Anne Pavlenko, Tanel Kaart, Lena Lidfors, David Richard Arney, Andres Aland

**Affiliations:** 1 Baltic Vianco Trading OÜ, Sänna Village, Rõuge Municipality, 66710 Võru County, Estonia; 2 Chair of Animal Breeding and Biotechnology, Institute of Veterinary Medicine and Animal Sciences, Estonian University of Life Sciences, Kreutzwaldi 62, 51006 Tartu, Estonia; 3 Department of Animal Environment and Health, Swedish University of Agricultural Sciences, P.O. Box 234, 53223 Skara, Sweden; 4 Chair of Animal Nutrition, Institute of Veterinary Medicine and Animal Sciences, Estonian University of Life Sciences, Kreutzwaldi 62, 51006 Tartu, Estonia; 5 Chair of Veterinary Biomedicine and Food Hygiene, Institute of Veterinary Medicine and Animal Sciences, Estonian University of Life Sciences, Kreutzwaldi 62, 51006 Tartu, Estonia

**Keywords:** Behaviour, Body condition score, Dairy cows, Lameness, Milk production, Transition

## Abstract

**Background:**

Transition of dairy cows from a tied to a loose housing system may affect their behaviour, health and production. Such housing system changes have become more frequent in Estonia but knowledge is lacking on how cows adapt to a new system. The aim of this study was to evaluate how cows’ behaviour, milk production and composition, and different aspects of their health changed after transition from tied to loose housing.

**Results:**

A herd of 400 dairy cows was moved to a new system on the same farm, so that effects of transport were not confounding factors. Behavioural observations were made for approximately 4 months following transition. Milk production data were recorded from 12 months before to 12 months after transition. Examination for skin alterations and cleanliness, as well as body condition scoring were carried out before transition, and thereafter monthly throughout the study. Significant effects on behaviour were observed just after the transition, with increases in the behaviour indicative of poor welfare, such as vocalisation and aggression, and decreases in those indicative of a good state of welfare, such as ruminating, resting and grooming. These effects were of short duration, with most returning to a steady state after the first week. Milk production declined already before the transition but fell significantly after transition, and this fall lasted longer in older cows. Likewise, somatic cell counts were higher in all cows following transition, but older cows were affected significantly more than cows in the first lactation. The frequency of lameness and skin alterations increased on average after transition. Body condition scores fell after transition but recovered by the second month. Therefore, there were adverse effects on the behaviour, health and production of the dairy cows transferred, although, apart from older cows, of short duration.

**Conclusion:**

The transition from tied to loose housing first had negative impacts on the welfare of the cows, although by the tenth day the behavioural indicators had returned to normal values. Impacts were more severe in higher parity cows, indicating that the change was more of a challenge for older cows.

The findings of this study suggest that animals’ behaviour and health should be more carefully observed within about 2 weeks after transition. It is quite likely that more and more farmers in Estonia and elsewhere will recognize the benefits of keeping their dairy cattle in loose housing, aimed at improving animal welfare and the value of the production chain.

**Supplementary Information:**

The online version contains supplementary material available at 10.1186/s13028-023-00690-1.

## Background

Tied housing is used worldwide [1]. However, there has been an increasing drive to move cows from tied to loose housing systems, and there is even legislation, e.g., in Norway, to enforce this [2]. Welfare has been observed to be improved in loose-housed compared to tie-housed cows [3]. Factors such as freedom of movement, choice of lying place, eating place, and social companions may be positive aspects of loose housing compared to tied housing [4]. However, other factors such as the level of milk production and certain health parameters may be affected in either a positive or a negative way, when the housing system is been changed from tied to loose housing. Presumably, cows are affected by stress during transition to a new housing system, but it is unknown for how long they are affected by the transition stress. Pavlenko et al. [5] recently showed that eating and ruminating behaviours were affected for the first 2 days, milk production was lower during the first month, and there were negative effects on reproduction after transfer from tied to loose housing. In that study, health measurements were not recorded.

It is of interest to investigate how long it might take for a large group of dairy cows introduced to a new environment, i.e., from tied to loose housing, to adapt to changes in social interactions and other behaviours, and to examine the effects of transition on milk yields and health. Additionally, how the transition of cows from tied to loose housing influences the occurrence of certain health- and welfare-related issues, e.g., skin alterations, lameness and other carpal and tarsal alterations, general cleanliness, and udder alterations, needs to be investigated. Of special interest is lameness and carpal and tarsal alterations as indicators of claw health. Claw diseases and mastitis are the main problems in loose-housed dairy cows, especially during the first lactation after the change in the housing system [6], and increasingly in many loose housing herds [7]. Claw disorders not only cause pain but also may lead to considerable economic losses to farmers as lameness is associated with reduced milk yield, decreased reproductive performance, and an increased risk of culling [8]. It also affects the behaviour of the cows, including during the milking process; Hassall et al. [9] found that lame cows entered the milking parlour significantly later than non-lame cows, and were more restless during milking.

The aim of this study was to evaluate how dairy cows’ behaviour, milk yield, milk composition, and certain health aspects were affected by transition from tied to loose housing. It was predicted that during the first days after transition the cows would have higher occurrence of behaviours indicative of stress, such as more frequent vocalisation, aggression, and abnormal behaviours, and lower occurrence of behaviours indicative of low or no stress, such as lying, eating, rumination, grooming. At the same time, animals transferred to loose housing would show more social behaviours. In addition, detecting animals in heat may become easier as they show a higher mounting activity. It was further predicted that stress would result in a decrease in milk production and an increase in somatic cell count, but that it would level out over a period of weeks. Cows were also predicted to initially have more challenges with body score condition, cleanliness, lameness, and with skin alteration on trunk, legs or udder after transition. Confirmation of these assumptions would provide valuable knowledge to be passed on to farmers planning to change from a tied to a loose-housed system.

## Methods

### Housing, management and animals

The study was performed in a dairy herd of approximately 400 cows. Before moving to the new loose housing system, all cows were permanently housed in an insulated cattle shed with a tied system. The cows were kept on a concrete floor. A combination of straw and peat was used as bedding in an amount of 2–3 kg per animal. All cows were milked while tied in their stalls twice daily, and were not pastured during the summer. The cows were fed twice a day with a total mixed ration (TMR) which remained available ad libitum. This was fed at a flat rate and consisted of grass hay, concentrate comprising brewers grains, yeast, palm fat, salt, and four blocks of mineral lick. Water was provided using a piping system with double-sided bowl dispensers. Cow footbaths were not used in tied housing. Half of the animals were Estonian Holstein and the other half of the Estonian Red breed.

The cows were neither separated based on breed during the transfer to the new system, nor distributed equally between the groups. Most of the cows transferred to loose housing were in their first or second lactation. At the first control milking in the new system, the number of cows per lactation number was: 1–152 (38.5%), 2–127 (32.2%) 3–56 (14.2%), and 4 or higher–60 (15.2%). Those considered unfit for the transfer, e.g., aged, diseased, in the post partum period and dry cows remained in the old tied system. The new, uninsulated loose housing cowshed that was built besides the old one, was designed for 400 milking cows. The new facility was divided into four sections, each with an area of 800 m^2^, equipped with 100 cubicles. Each section had concrete flooring and cubicles were covered with rubber mats (DeLaval, Sweden); no bedding material was used. Walkways to the milking area were covered with rubber mats. Manure was removed at two-hour intervals using an automatic scraper. Cows returning from milking walked through two dry footbaths containing a disinfectant based on copper and zinc compounds.

**Fig. 1:**
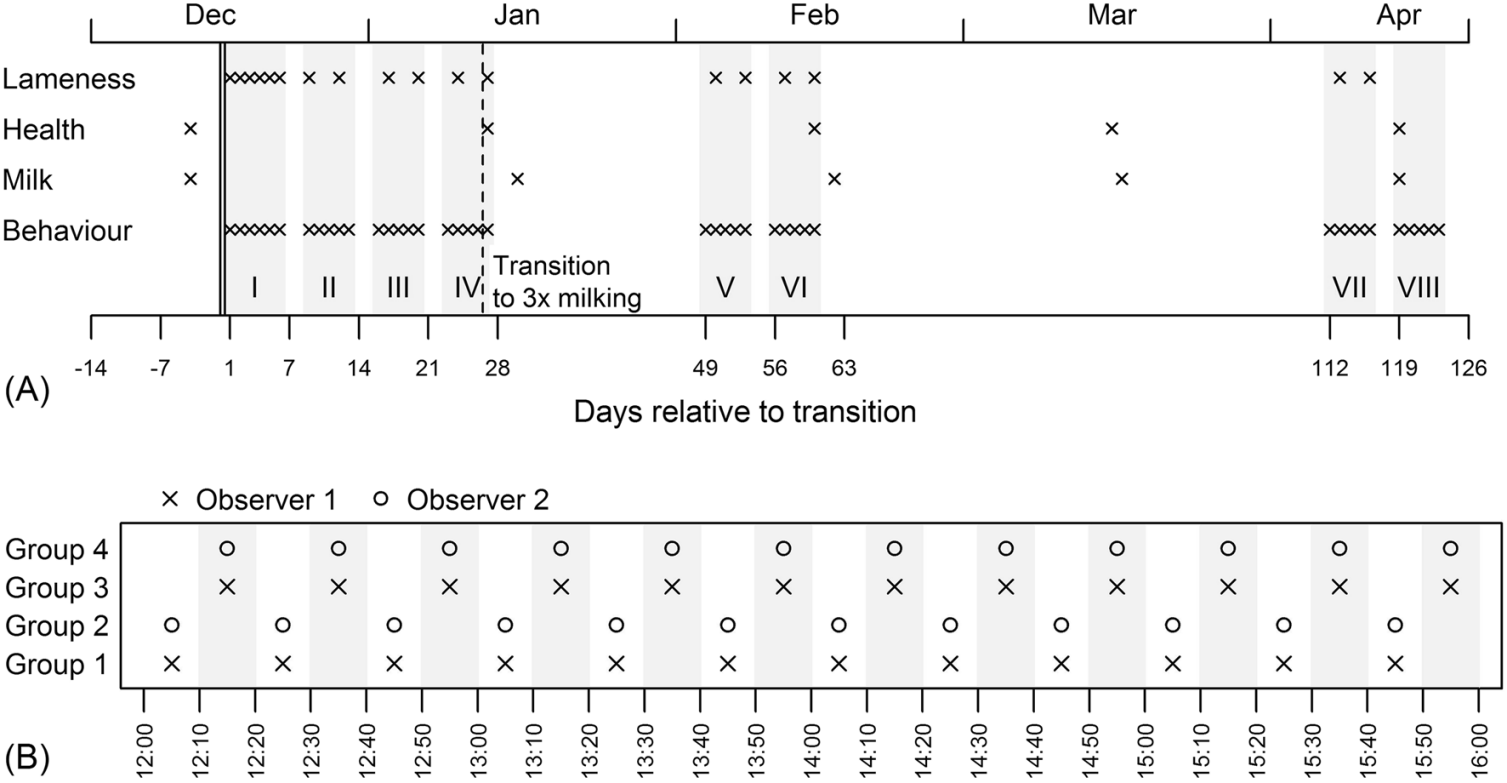
Sampling scheme. **A** The transition time is denoted with a double vertical line, crosses mark sampling days; grey bars with roman numerals denote behavioural and lameness observation periods, dotted line the time of milking change. Milk denotes test-day milk samples and health denotes observations of body condition score, skin alterations, carpal and tarsal alterations and udder alterations, and general cleanliness at the cow level. Lameness and behaviour were registered at group level. **B** Time distribution of behavioural observations per day for four groups of cows (the cows were fed twice a day at 8.30 and 12.00 h, and milked initially twice a day at 4.00 and 20.00 h, and subsequently three times per day at 4.00, 12.00 and 20.00 h)

As the new building was constructed near the old one, cows were walked to the new cowshed. Cows were moved to the new loose housing facility over the course of a 5-day period from the end of December to the beginning of January. A total of 230 cows were moved on the first day, followed by around 20 cows each subsequent day. By the end of the first week, 350 cows had been moved to the new facility. The cows were grouped according to their lactation stage and milk yield: one group for open (recently calved and not pregnant) cows, two groups for those at peak lactation stage, and one group for pregnant cows with low milk yield and/or approaching dry-off. Frequent relocation of cows was avoided, but due to changes in lactation stage and transferring/returning of diseased and recovered cows, the group sizes were not determined (on an average, each of the first three groups comprised 100 cows, and the fourth one 80 cows). As decided by the farm management, during the first month after transition, cows were milked twice a day at 4:00 and 20:00, and subsequently (Fig. 1A) three times a day at 4:00, 12:00 and 20:00. The milking took place in a 2 × 10 parallel milking parlour (DeLaval, Sweden) with cows spending an average of 75 min in the waiting area. Just as before the transition, the cows were fed twice a day (at 8:30 and 12:00) with the same total mixed ration (TMR) available ad libitum. TMR consisting of grass hay, and a concentrate comprising brewers’ grains, yeast, palm fat, and salt, was provided from the feed stall of the feeding passage. A separate container with four blocks of a mineral lick was placed next to the feeding area and made accessible to all groups. Water was available ad libitum from two water troughs of different size (2.5 × 0.6 m and 1.4 × 0.6 m) per lactation group. Due to the depreciation of the old tied facility, the farmer had planned a transition anyway, thus the designing and building of a new cowshed was not a part of our research plan. As the animals were only observed and no management changes were made due to the study, a submission to the ethical committee was not required [10].

### Behavioural observations

Behavioural observations were started 1 day after moving the first cows, and were carried out according to the plan shown in Fig. 1A. Observations were made at group level for 4 h per day from 12:00 to 16:00, between milking, separately for the four groups of cows. Two observers made and recorded behavioural observations simultaneously according to the plan presented in Fig. 1B. The observers had completed studies in veterinary medicine or animal science. They had taken a course in animal behaviour and welfare where the design of this kind of studies was taught, and had undergone a 3-day period of training in carrying out observations and recording them. Their knowledge and skills were checked and approved before the start of the study. However, the inter-observer reliability was not evaluated. At all times, the observers stayed outside the area where cows were housed. The distance between an observer and an observed group was approximately 3–7 m. Instantaneous recordings were made at 10-min intervals as follows: at the beginning of each 10-min period, the total number of cows in the group and the number of cows in the group adopting any of the three body positions, seven general behaviours, four social behaviours and three abnormal behaviours were recorded (Table 1). For each group, 12 instantaneous recordings at 10-min intervals per day on 41 observation days were made, which equals 492 interval recordings in total. Therefore, a total of 1,968 recordings were made for all four groups of cows. Due to unexpected changes in milking times, mixing of groups or other events, or when the observed group of cows was not in their section, data from 392 (19.9%) 10-min interval recordings were not available for statistical analyses. For each observation period and group, potential disturbing factors (tractor, other visiting people) were recorded to consider their potential confounding effects in the statistical analyses.

**Table 1:** Body positions and behaviours recorded during direct observations, and their definitions

Behaviour	Definitions
Body positions	
Standing	Standing with all four hooves on the floor
Lying	Without support of any leg and with the belly in contact with the floor
Walking	Placing one hoof forward and down on the floor thus moving the body in the walking or eating area
General behaviours	
Eating	Head down close to TMR or chewing while standing at the feed face
Drinking	Head down close to water or muzzle in contact with water
Ruminating	Regurgitating or chewing on regurgitated bolus
Sleeping	Lying down on the side or on the belly, not ruminating, head up or turned to the side or stretched out forward, eyes closed or half closed
Grooming	Tongue repeatedly in contact with the cow’s own body
Exploring	Nose or tongue in contact with, or within 10 cm of, fittings
Looking	Eyes directed towards a certain point and ears turned forward in the same direction
Social behaviours	
Social licking	Tongue repeatedly in contact with the body of another cow
Mounting	Placing the front legs and breastbone on the back of another cow
Pushing and butting	Putting the head against the body of another cows’ head or sides and using some force to move that animal
Vocalising	High-pitched open mouth calls
Abnormal behaviours	
Tongue rolling	Rolling movements performed by the tongue not connected to eating, drinking or licking
Nose pressing	Pressing the nose against farm equipment for a period of more than 10 s

### Production

Milk yield (kg), milk fat and protein (%), and somatic cell counts (SCC) were recorded once per month (monthly test-day milking) by Estonian Livestock Performance Recording Ltd. The test-day milking of the transferred cows was made 12 months before and 12 months after transition. The last test-day milking in the old cowshed was performed 4 days before moving, and the next one 33 days after moving to the new loose housing unit (Fig. 1A). The mean number of test-day milkings per month was 379.4, with a range from 274 to 438. To achieve a symmetrical distribution, the initially right-skewed somatic cell counts were transformed to the somatic cell score (SCS) according to the formula SCS = log_2_(SCC/100,000) + 3.

### Health and body condition

Four days before the cows were moved to the new loose housing unit and behavioural observations began, and after transition monthly throughout the study, some health and body condition parameters were checked and recorded in all cows (Fig. 1A). Three trained persons performed observations from a distance of 3–7 m from the alley of the following health recordings: skin alterations on the trunk, carpal and tarsal alterations, udder alterations, and general cleanliness. The observers, in line with the previously agreed methodology, carried out scoring simultaneously. The scales and definitions of each category for general health recordings are shown in Table 2. Body condition was scored using the method developed by Ferguson et al. [11] in five-point scale with 0.25 increments. A modified scale based on Sprecher et al. [12] was used for lameness scoring: cows with normal walk, no arched back and normal standing posture were scored 0; cows with normal gait but with shorter steps, arched back, changed position of the feet or raising and keeping one hoof raised when standing were scored 1; cows with arched back while walking and standing, difficulties in walking, asymmetric gait or difficulties while standing were scored 2. Lameness scoring was conducted six times during period 1 and twice per period for periods 2–7. As the single cows were not identified, only the number of cows per group and per breed that received different lameness scores was used for statistical analysis.

**Table 2:** Description of health recordings

Scoring	Description
Skin alterations	
0	Skin healthy, no visible wounds, no external parasites
1	Some old wounds or scratched areas, mild external parasite lesions
2	Fresh wounds, swollen areas, severe external parasite lesions
Carpal and tarsal alterations	
0	No visible lesions on carpal or tarsal areas. Skin normal in this area, no swollen joints
1	Some old visible wounds or scratched areas on tarsal or carpal areas. Joints not swollen
2	Fresh wounds on tarsal or carpal areas, joints may be swollen, cow may raise her hoof (she avoids stepping on it)
General cleanliness	
0	Clean: coat clean, some small dirty spots on legs
1	Medium: legs dirty, some spots also on flanks
2	Dirty: legs, udder, flanks are very dirty
Udder alterations	
0	No obvious physical changes
1	Udder problems (udder lesions, swellings, wounds)

### Statistical analyses

Statistical analyses were performed using SAS 9.4 (Statistical Analysis System Inc., Cary, NC, USA) and figures constructed using R 4.2.0 (R Foundation for Statistical Computing, Vienna, Austria). The experimental unit in production data analyses was the animal, in behavioural data analysis the group of animals, and in health and body condition’ data analyses the breed subgroup of the observational group of animals. The changes in cows’ body positions and behaviours (percentages), and in occurrence of different lameness score values, skin alterations, carpal and tarsal alterations and udder alterations after transition, were studied fitting generalized linear mixed models with logistic link function using SAS procedure GLIMMIX. The body condition scores and cleanliness scores after transition, as well as milk production and milk quality traits 1 year before and 1 year after the transition, were analysed with general linear mixed models using SAS procedure MIXED. In the models, the fixed effects of time, group (behavioural and lameness data), external disturbing factors (behavioural data), lactation month (production data) and breed (health data) and the non-zero covariance of model errors corresponding to repeated measurements of the same animal or group of animals, as well as the random effect of observer in case of multiple observers, were considered. Results are presented as least square means (*alias* marginal means) with model-based standard errors. As there were no differences for any of the behaviours between periods 7 and 8 at 4 months after transition, and periods 5 and 6 at 2 months after transition (all P > 0.05), only results for the first six observational periods covering the first 2 months after transition are presented. For more details about fitted models, see Additional file 1. The time periods were compared with appropriately defined contrasts and the Tukey post-hoc test for pairwise comparisons. Results were considered statistically significant at P ≤ 0.05.

## Results

### Behaviour

**Fig. 2:**
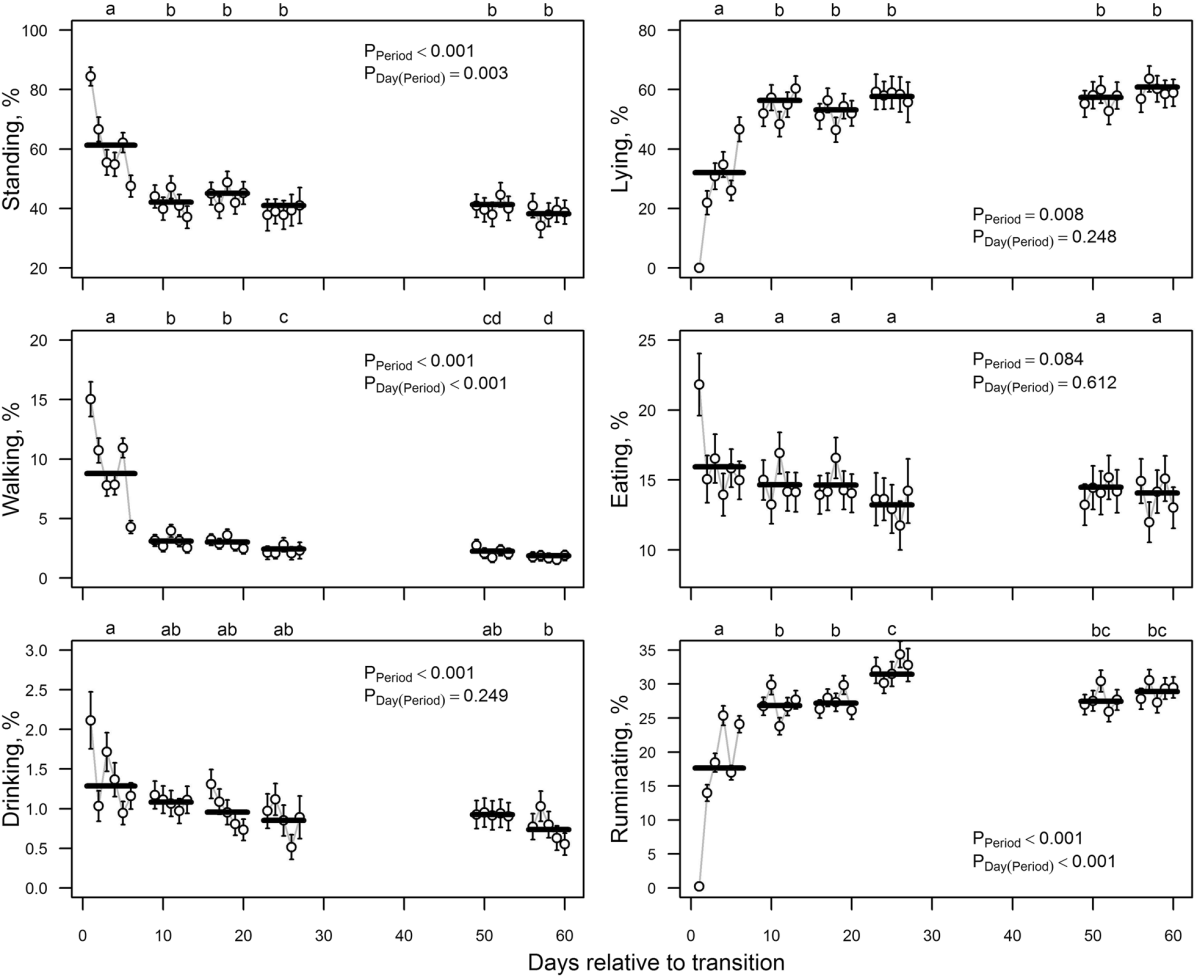
Estimated percentage (± standard error) of body positions and more frequent general behaviours on different days (dots with error bars) and periods (bold horizontal lines). P-values indicate the significance of period and day nested to period effects according to the logistic model, also considering the effects of external disturbing factors, group and group-by-period interaction, and random effect of observer (except walking and drinking) and the first order autoregressive co-variation structure of model errors corresponding to observations made in the same group on the same day. Letters above the figures denote statistical significance of between periods differences (periods without a common letter are statistically significantly different: Tukey *post-hoc* test for pairwise comparisons, P < 0.05)

For all three body positions, the first period was significantly different from the subsequent periods: cows were observed to be standing and walking more and lying less in the first period (Fig. 2). The between-days variation within period was marginal for all other periods except the first. On the first day of the first period, cows mainly stood (84.4%) or walked (15.1%). Over the next 4 days, lying was recorded for 21.9–34.8% of cows, and by the sixth day after transition, the standing, lying and walking proportions were already close to the proportions characteristic of the subsequent periods (Fig. 2).

**Fig. 3:**
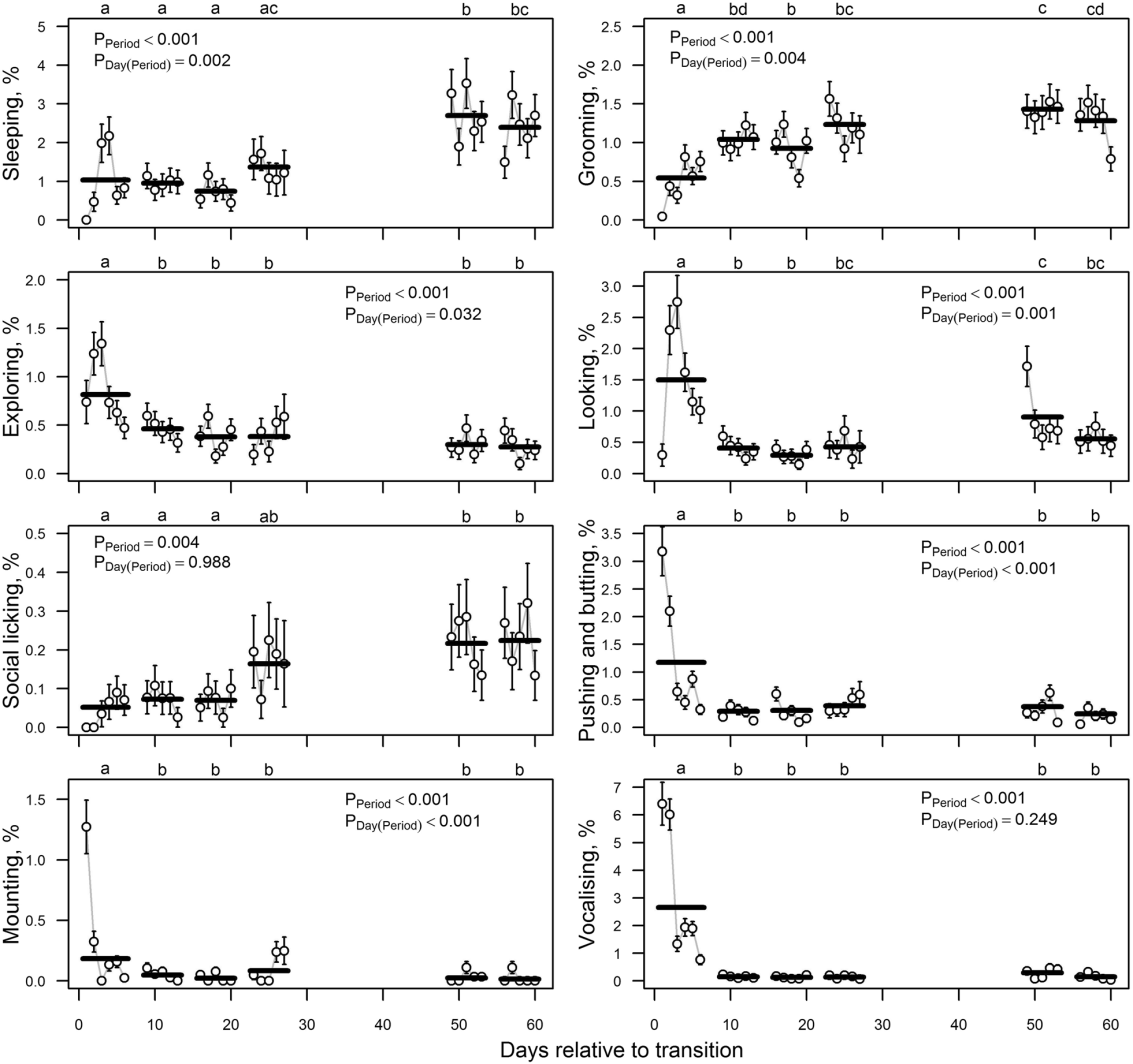
Estimated percentage (± standard error) of less frequent general behaviours and social behaviours on different days (dots with error bars) and periods (bold horizontal lines). P-values indicate the significance of period and day nested to period effects according to the logistic model, also considering the effects of external disturbing factors, group and group-by-period interaction, and the first order autoregressive co-variation structure of model errors corresponding to observations made in the same group on the same day. Letters above the figures denote statistical significance of between periods differences (periods without a common letter are statistically significantly different: Tukey *post-hoc* test for pairwise comparisons, P < 0.05)

In the first period after transition, cows had higher proportion of eating and drinking and lower proportion of ruminating compared to the subsequent periods. However, this difference was significant only for reduced rumination. The between-days variation within period was the highest in the first period; on the first day after transition, cows had a much higher proportion of eating and drinking than on subsequent days (Fig. 2). In the first period after transition, exploring and looking had significantly higher proportions and grooming had a significantly lower proportion (Fig. 3). Similarly, to other general behaviours, the within-period variability was highest in the first period. However, the increase in exploring and looking relative to the other days was not on the first, but on the second and third days after transition (Fig. 3). The only general behaviour for which a pattern was not stabilised within 1 month was sleeping (Fig. 3). During the first four periods (the first month) after transition, sleeping was recorded in only about 1% of the observations, which was half that for the periods 5–6 (Fig. 3).

Of the four studied social behaviours, patterns of mounting, pushing and butting, and vocalising were similar to each other (Fig. 3). The behaviours had a significantly higher proportion in the first period, showing especially high proportions in the first 2 days; they stabilised by the end of the first week after transition. The pattern of social licking was different: in the first three periods, half the amount of social licking was observed compared to the subsequent periods (on the first 2 days after transition, no social licking was recorded) (Fig. 3). The significantly higher proportions of social licking compared with the first period was achieved only in the 5th and 6th periods (Fig. 3). Of the recorded abnormal behaviours, there was slightly more tongue rolling in the first weeks after transition; of 14 observations, a total of eight instances of tongue rolling were recorded during the first four periods, six of which occurred during the first two periods. Contrarily, nose pressing was more frequent in the later periods—of 11 observations in total, only two were recorded during the first four periods, another two during the 5th and 6th period, and the remaining seven during the 7th and 8th observational periods.

### Production

**Fig. 4:**
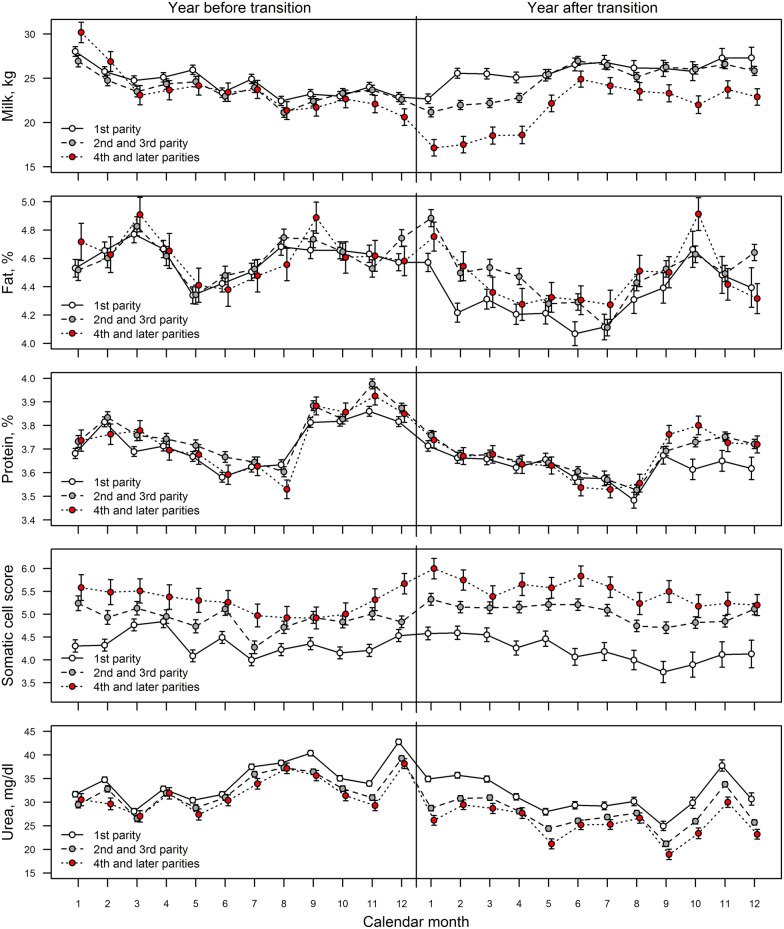
Monthly least square means (± standard error) of cows’ milk production and milk quality traits 1 year before and 1 year after the transition of cows, with parity. Calculations according to a general linear mixed model considering fixed effects of parity, lactation month and calendar month by calendar year by parity interaction, and non-zero co-variation of model errors corresponding to recordings of the same cow. The somatic cell score (SCS) was calculated as SCS = log_2_(SCC/100,000) + 3, where SCC denotes somatic cell count

Results indicate that the milk production decreased and the SCC scores increased already before the transition, and the same trends continued after transition, especially in older cows (Fig. 4). The estimated differences (± standard error) between test-day milk yields in the last month before and the first month after the transition were −0.19 kg (± 0.63, P = 0.770), −1.44 kg (± 0.59, P = 0.015) and −3.46 kg (± 1.06, P = 0.001) for the 1st, 2nd–3rd, and 4th and higher parities, respectively. For older cows, it took 5–6 months to achieve their pre-transition production level, while the milk production of the first parity cows increased after 1–2 months from transition. The estimated differences (± standard error) between test day somatic cell counts in the last month before and the first month after the transition were 0.05 (± 0.17, P = 0.782), 0.49 (± 0.16, P = 0.002) and 0.33 (± 0.28, P = 0.244) for the 1st, 2nd–3rd, and 4th and higher parities, respectively. The milk fat percentage increased slightly in the first month after transition (for all parity groups P > 0.05), but dropped rapidly after that (P < 0.001 for the 1st, and 2nd–3rd parities and P = 0.113 for the 4th and higher parities). The milk protein percentage decreased after transition (P < 0.001 for all parity groups) following the negative trend, which began 2 months before transition and continued 8 months after transition. The milk urea content decreased significantly after transition (P < 0.001 for all parity groups).

### Health and body condition

**Fig. 5:**
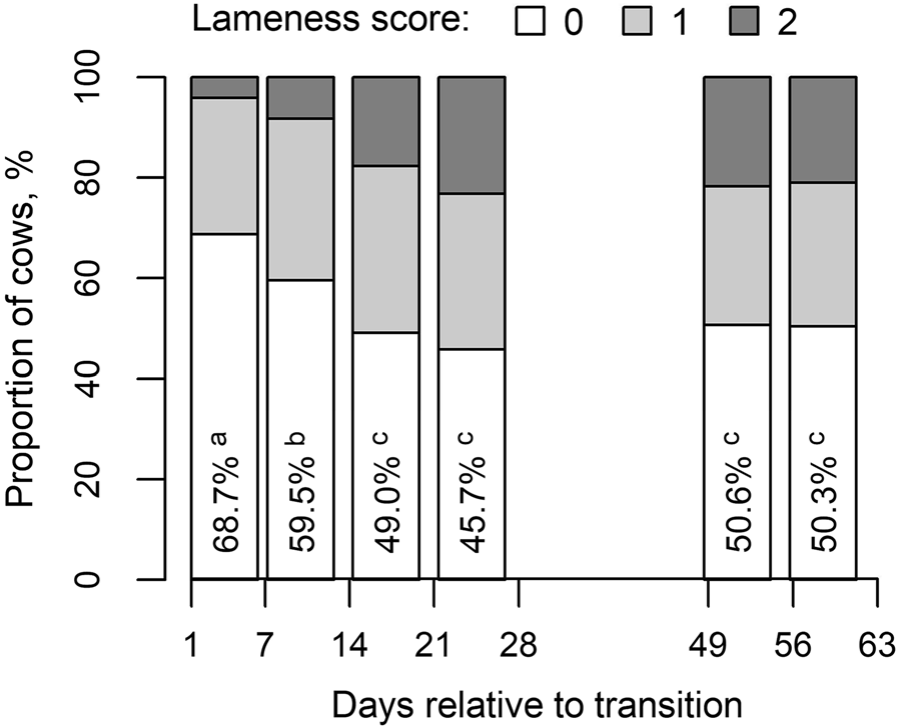
Proportions of cows without lameness (score 0), with medium lameness (score 1) and with severe lameness (score 2) depending on observational period. Different letters indicate significantly different periods (Tukey *post-hoc* test for pairwise comparisons following logistic regression analysis, P < 0.05)

While in the first week after the transition 68.7% of cows showed no lameness and 4.2% severe lameness (Fig. 5), a week later, these proportions had changed and higher lameness was recorded in 8.3% of cows, whereas 59.5% of cows showed no lameness. In the third week after the transition only 49.0% of cows showed no lameness and 17.8% of cows were diagnosed with severe lameness. These proportions remained similar throughout the following observational periods, including the 7th period 4 months after the transition when 48.8% of cows showed no lameness, and severe lameness was recorded for 20.6% of cows. The proportion of cows with medium lameness varied between 27.4 and 34.1%. Changes in lameness depended neither on the breed nor the group of cows (breed-by-period and group-by-period interaction effects were not significant for any of the lameness categories).

**Fig. 6:**
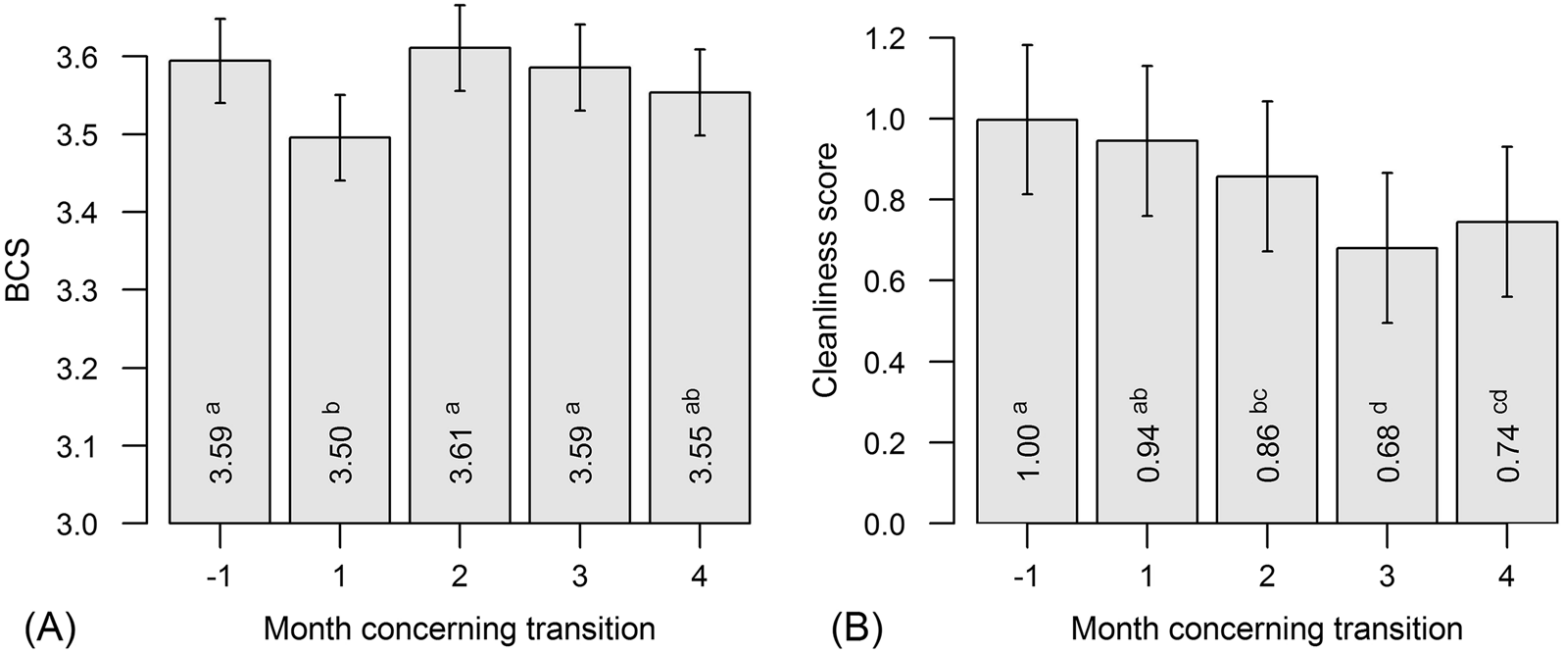
Least square means (± standard error) of cows’ **A** body condition scores (BCS) and **B** cleanliness scores at different months relative to transition. Calculations derived from a general linear mixed model considering fixed effects of month, breed and month by breed interaction, and random effect of observer; means without a common letter are statistically significantly different (P < 0.05, Tukey *post-hoc* test for pairwise comparisons)

The mean body condition score (BCS) was, 1 month after transition, significantly lower (Fig. 6A) compared to that 1 month before transition (P < 0.001). However, 2 months after transition the body condition of cows was back at the pre-transition level. The mean BCS for Estonian Holstein cows was 0.08 points lower compared to those for the Estonian Red cows (P < 0.001), but there was no month-by-breed interaction effect (P = 0.509).

Cleanliness improved after cows’ transition (the lower the score the cleaner the cow). While before transition the mean cleanliness score was 1.00, 1 month after transition the mean score was 0.94 and this decrease continued (Fig. 6B). The mean cleanliness score of Estonian Holstein cows was 0.09 points higher (i.e., they were dirtier) than the mean cleanliness score of the Estonian Red cows (P = 0.002). There was also a significant month-by-breed interaction effect (P < 0.001); the improvement in cleanliness after transition was greater among Estonian Red cows (from 1.08 before transition to 0.66 4 months after transition). Before transition, Estonian Holstein cows had a mean cleanliness score of 0.92, and 4 months after transition the mean score was 0.83.

**Fig. 7:**
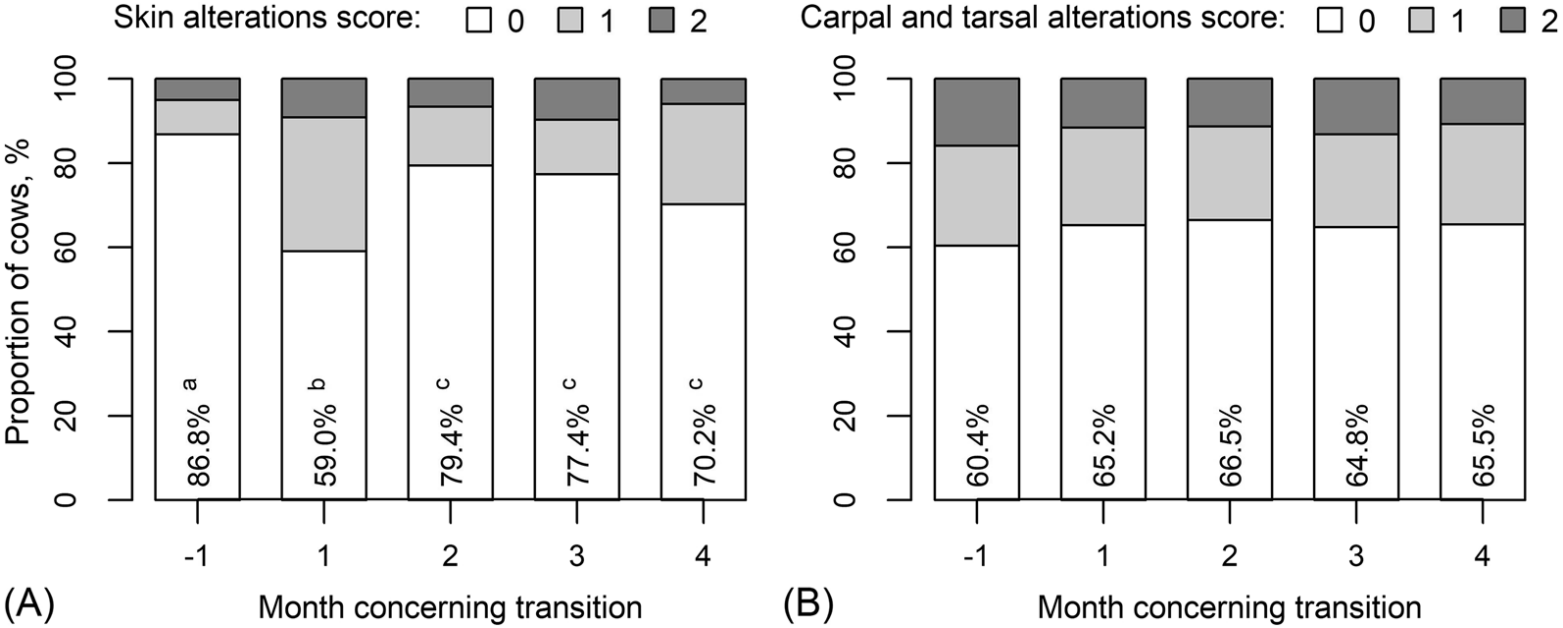
Proportion of cows with different **A** skin alterations scores and **B** carpal and tarsal alterations scores at different months concerning transition. Numerically are presented proportions of cows with zero-score, different letters indicate statistically significantly different months (Tukey *post-hoc* test for pairwise comparisons following the logistic regression analysis, P < 0.05; there were no statistically significant differences between months in carpal and tarsal alterations scores)

For skin alterations there was a significant association with month after transition (P < 0.001). One month after transition the proportion of cows without skin alterations dropped from the pre-transition level of 86.8–59.0% (P < 0.001) (Fig. 7A). In later months, the proportion of cows without skin alterations increased significantly but remained significantly lower than before transition. The percentage of cows with skin alterations score of 2 was more stable and remained within the interval 5.1–9.7%. There was also a significant difference between breeds (P = 0.007). Among Estonian Reds, the number of cows with skin alterations was 5.8% higher than among Estonian Holsteins. Estonian Red cows also had a higher number of skin alterations scores of 1 and 2. However, no month-by-breed interaction was found (P = 0.284).

The proportion of cows with carpal and tarsal alterations score of 0 increased after transition within 1 month by about 5%, from 60.4% to 65.2%, and remained at around this higher level for the subsequent study months (Fig. 7B). However, the effect of month was not statistically significant (P = 0.099). In addition, there was no effect of breed (P = 0.447) nor month-by-breed interaction (P = 0.937).

The proportion of cows with udder alterations decreased from 11.3% before transition to 9.0% and 7.5% one and 2 months after transition, respectively (P = 0.074). The proportion of cows with udder alterations was 4.3% higher among Estonian Holstein cows compared to Estonian Red cows (P = 0.003) and this difference remained the same throughout the study. There was no month-by-breed interaction effect for udder alterations (P = 0.790).

## Discussion

### Behaviour indicators

Standing and walking, which might indicate discomfort at abnormally high rates [13], were reduced from the immediate post-transition scores to stable scores by day 10, although this might be an artefact of the higher lying rates observed by day 10, the two complementing each other. A lack of comfort may result in reduced time spent lying, and an increase in time spent standing without eating [14, 15]. Lying is a highly motivated behaviour for dairy cows and lying time has higher priority than eating time and social contact [16], and deprivation of lying increases behavioural signs of discomfort [17]. The fact that the cows spent less time lying in the first 10 days after transition may indicate a state of unease in these cows.

Feeding (by day two) and drinking (by day five) were both at stable levels soon after transition, thus effects on these behaviours were therefore of short duration. The body condition score changes appear to reflect these observations of feeding behaviour; both decreased after transition but then recovered shortly thereafter. However, ruminating took a longer time to reach a steady level, at least for the first 6 days. This is different from an earlier study by Pavlenko et al. [5], where total rumination did not differ between days after transfer from tied to loose housing. Loberg et al. [18] concluded from their study that for tie stall cows, eating, ruminating and lying were the only activities available, while those provided with outdoor access for exercise daily, 2 days a week, or 1 day a week, had more diverse movement opportunities. We assume that in our study, reduced rumination indicated that the cows introduced to loose housing initially experienced alternative movement options in their new environment as temporary factors affecting rumination time.

However, in a previous study, and considering body position, cows ruminated while standing significantly more for the first 3 days compared to days 29–31 after transfer from tied to loose housing [5]. Pavlenko et al. [13] found that rumination while standing was higher in dairy cows with digital dermatitis, and tended to be higher in cows with sole ulcers, indicating that they had problems with lying down. However, Walker et al*.* [19] found that lame cows spent less time standing while ruminating as compared to healthy cows. Österman and Redbo [20] showed that if cows experience more discomfort, they stood more while ruminating, and had shorter and fewer lying bouts. In this study, whether cows were standing or lying while ruminating was not recorded, so it is only possible to draw conclusions based on the total proportion of cows ruminating.

Loberg et al. [18] suggested that exploratory behaviour to a larger extent is motivated by the new environment, and that part of the motivation for walking, trotting and playing in adult dairy cows is internal, and builds up with time of confinement. Veissier et al. [21] noted that when the cows that had been housed in tie-stalls without exercise opportunities were released into an arena, an increase in locomotion was observed. However, they found it unlikely that the greater locomotion was due to a greater tendency to explore the environment, as the animals were more or less familiar with their surroundings. In our study, however, the exploratory behaviour was higher on days 2 and 3 after transition, and then decreased to a relatively steady state by day 10 post-transition. This could be explained by the increasing familiarity of the environment and the consequent reduced motivation over time for investigating the environment. Pavlenko et al. [13] found that cows with claw problems explored farm equipment and/or the ground more than healthy cows, which was interpreted as the cows searching for a suitable place to lie down. The tied dairy cows, exercised once or twice per week, showed more exploratory behaviour during exercise than those exercised every day [18]. This was probably caused by a novelty effect when there was an interruption for some days in the exercise. When dairy cows that were kept loose permanently on deep bedding with access to a yard and pasture were compared to tie-housed cows, exploratory behaviours were 2–3 times higher in the tie stall [22].

Grooming was observed infrequently in the first weeks, but it gradually increased by day 20 when it seemed to stabilise. Pavlenko et al. [5] also found that cows groomed themselves less when moved from a tie to loose housing. Despite this, the animals were cleaner after transition as the brushes in the loose housing and the possibility to choose a clean lying place may have helped keeping the cows cleaner. Dairy cows kept permanently on deep bedding with access to a yard and pasture were found to perform less grooming than tied cows [23]. On the other hand, novel food and an unfamiliar person caused cows to increase their self-grooming [24]. The interpretation of the grooming behaviour could be that when cows are moved to a new system they focus less on keeping themselves clean [5], whereas when kept permanently tied or when something novel happens, grooming becomes more frequent.

The behaviour defined as ‘sleeping’ in this study took longer to recover, remaining low until day 50 after transition. As there were no individual recordings of sleeping, but only the number of cows showing this behaviour determined by instantaneous recordings, it is difficult to compare these results with other studies. In this study, sleeping was defined as “lying with head up or turned to the side or stretched out forward with eyes closed or half-closed”. In other studies, a more strict definition of sleeping has been used, and in some studies complemented by EEG [25, 26]. Therefore, the number of cows that were sleeping in this study may have been overestimated.

Behaviours that might be expected to be more pronounced in poor welfare conditions, such as aggression and vocalisation, declined to stable rates by around day 10 after transition.

Aggressive behaviour is thought of as being motivated by the presence of an opponent and the animal’s past history of encounters [27]. We suggest that the cows grouped together after transition might have regarded each other as opponents for some time, until they became more familiar with each other. The mixing of cows probably caused the high levels of pushing and butting during days 1–2. This fits well with the lack of social licking in these cows. In a stable herd social licking occur predominantly between cows that have a social bond with each other [27]. The occurrence of mounting behaviour was very high on the first 2 days after transition, and decreased significantly later. It may have been that sexual cycles among these cows were somewhat in synchrony. However, at this time of heightened distress, mounting might have been performed more as a displacement behaviour.

Vocalisation has been considered a welfare indicator, and gives us information about an animal’s internal state, if we can interpret these vocalisations correctly [28, 29]. Pavlenko et al. [5] found that cows vocalized more after transition from a tied to loose housing, and they vocalized more if some external disturbances appeared, e.g., feeding took place or other animals or humans were passing by. Grandin [30] assumed similarly that vocalisations do occur more often when a cow experiences an aversive event compared to less aversive ones. We suggest that an increase in vocalisations during the first days under loose-housing conditions are signs of distress or discomfort caused by moving.

There were too few recordings of abnormal behaviours such as tongue-rolling and nose-pressing to be able to test them statistically. However, there were slightly more observations of tongue-rolling in the first weeks, and more nose-pressing during the last weeks of observation. Tongue rolling [31] and nose-pressing [32] are recognised stereotypies in cows. Stereotypic behaviours are recognised cross-disciplinarily as being related to stress in animals [33]. Tongue-rolling and nose-pressing may be regarded as the individuals’ strategies to cope with stressful events. Nose-pressing have been suggested as a way for cows to re-direct pain [34]. Holstein cows naturally infected with bovine lentivirus 1 bovine immunodeficiency virus (BIV) and other infections were observed to occasionally show head or nose pressing postures [35]. Nose-pressing cows were found to have a significantly lower heart rate than non-nose-pressing cows and a higher parasympathetic activity when nose-pressing during milking, measured by their heart rate variability [36]. Dairy cows with digital dermatitis or sole ulcers showed occasional nose-pressing, but also healthy control cows showed nose-pressing so it could not be linked directly to the disease [13]. It has been shown that tongue-rolling is affected by breed (Jersey cows show more than Jersey-Holstein crosses), parity (second-parity and older cows show more) and lactation stage (tend to increase in early lactation and decrease in late lactation) [37]. In this study, there were too few recordings of tongue-rolling to make any statistical comparisons.

Behavioural results taken as a whole provide evidence that the cows’ welfare was first affected negatively by the transition to the new housing, but returned to basal stable levels after around 10 days in the new housing environment. In terms of the farmer's expectations regarding the transfer, such positive behavioural indicators like species-specific and social behaviour, including freedom of movement and choice of lying and eating places, became more apparent after 7‒10 days in a novel loose housing.

### Production

It was expected that the transition would likely be a stressor for cows and would decrease the milk production due to more movements and interactions with other cows. Stress is known to have a negative effect on milk production levels, both in relation to stockperson behaviour and attitude [38, 39], and increased environmental heat [40, 41]. The drop in production levels subsequent to transition was therefore not surprising. Evidence from production parameters seems to identify a different response to transition depending on the age of the cows, with older cows appearing to be more negatively affected than younger cows, although all cows suffered a transitory drop in production, confirming recent findings elsewhere [5]. Cows with a parity of greater than 3 did not return to pre-transition production values until the 5th–6th months post-transition, while first lactation cows had returned to pre-transition levels by the second month of transition. Intermediate cows also showed an intermediate rate of return to previous milk yields. It is conjectured that the older cows were less able to adapt to new circumstances than the younger cows. This finding is quite new, as behaviour studies usually balance for parity. A recent study [42] compared lying behaviour of primiparous and multiparous cows on introduction to a new group, and found that subsequent lying times were shorter in the primiparous cows, but this work only considered lying while other factors may also have affected this.

Raised somatic cell counts have been known for some time to be associated with stress [43], and have been used as a measure for evaluating the effect of stressors such as heat in cows [44]. In this study, the older cows had consistently higher somatic cell counts than younger cows in the months subsequent to transition, while this age difference was not observed prior to transition. There is therefore indication from somatic cell counts that the older cows were stressed more by transition than the younger cows. Milk fat (reduced in stressed cows [45]), protein (reduced in stressed cows [46]) and urea (reduced in stressed cows [46]) showed similar patterns of decline immediately following transition, followed by recovery to pre-transition levels, whereas in these cases no evidence of the effect of parity was found.

### Health

Straightforward interpretation of the findings related to the health status of cows was somewhat impaired due to the gross and sometimes inadequate observations performed from the distance of 3─7 m. However, as the observations were unbiased, we believe that the results are reliable enough to be used.

Incidences of lameness rose in the second week, and rose again in the third week, thereafter remaining unchanged. Although not significant, some evidence of higher rates of carpal and tarsal alterations among the cows following transition supports the concomitant increase in lameness. The causes of lameness are numerous and disparate, and this increase could have been the result of any of these, including unfavourable lying conditions or quality [47], poorer unaccustomed flooring [48], infections [36], poor hoof treatment or prophylaxis [49], inadequate response to pathogens as a result of stress [50], or a combination of the factors mentioned. It is impossible to exclude these causes, but it is nevertheless evident that lameness became more of a frequent problem for the cows after transition. The newly loose-housed cows improved their cleanliness scores throughout the duration of the trial; loose-housed cows have been found to be cleaner than are those kept on straw [51]. In addition, rotating brushes had been provided for the cows in the new housing. The incidence of skin alterations increased after transition, and there was a significant difference between breeds. This could have been due to some particles or objects that caused injuries, the reason that the cows were lying outside the cubicles, or that aggressive interactions caused slipping. There was a slight decrease in udder alterations following transition, although there was a difference between the two breeds, with the Estonian Red cows having fewer problems than the Holsteins. In fact, this is characteristic of lower-yielding breeds [52], which the Estonian Red is compared to the Estonian Holstein [53].

## Conclusions

The transition from tied to loose housing initially had negative impacts on the welfare of the cows, although by the tenth day relevant behavioural indicators had returned to normal values. Impacts were more severe in higher parity cows, thus indicating change was more of a problem for older cows. The findings of this study suggest that animals’ behaviour and health should be more carefully observed for about 2 weeks after transition. Despite the initial negative impact of the transition on cows’ welfare, the general positive effect of loose housing expressed e.g., by the freedom and ability of movement, choice of lying and eating place as well as of social companions clearly show its advantages over tied housing. It is quite likely that more and more farmers will recognize the benefits of loose housing vs tied housing in terms of animal welfare and the increased value of the entire production chain.

## Supplementary Information

**Additional file 1.** Detailed description of fitted statistical models.

## Data Availability

The datasets used and/or analysed during the current study are available from the corresponding author on reasonable request.
