# Supplementary material for: Changes in dairy cows’ behaviour, health, and production after transition from tied to loose housing
Source: Acta Vet Scand. 2023 Jun 30;65:29. doi: 10.1186/s13028-023-00690-1 (PMC10314408; doi:10.1186/s13028-023-00690-1)
Supplement: Supplementary file 1 — Additional file 1. Detailed description of fitted statistical models. [file 13028_2023_690_MOESM1_ESM.docx]

***Additional file 1. Detailed description of fitted statistical models***

Statistical analyses were performed using SAS 9.4 (Statistical Analysis System Inc., Cary, USA) and figures were constructed using R 3.3.3 (R Foundation for Statistical Computing, Vienna, Austria).

The experimental unit in cows’ body positions and behaviours data analyses was the group of cows who were observed repeatedly. For each group, there were 12 instantaneous recordings at 10-minute intervals per day on 41 observation days, which was equal to 492 interval recordings in total. Therefore, there were for all four groups of cows a total of 1,968 recordings. Due to unexpected changes in milking times, mixing of groups or other events, or when the observed group of cows was not in their section, data from 392 (19.9%) 10-minute interval recordings were not available for statistical analyses. The changes in percentage of cows’ body positions and behaviours after transition were studied fitting the generalized linear mixed models with logistic link function considering fixed effects of period *P_i_*, day nested to period *D_j_*(*P_i_*), external disturbing factors *F_k_*, group *G_l_* and period by group interaction *P_i_***G_l_*, random effect of observer *O_m_*~N(0,*σ*^2^*_O_*) (except walking and drinking where the models did not converge) and the first order autoregressive co-variation structure of model errors corresponding to observations made in the same group at the same day, cov(*ε_ijklmn_*,*ε_i’j’k’l’m’n’_*) = *σ*^2^**ρ*^|^*^n^*^-^*^n’^*^|^, if *i*=*i’* and *j*=*j’*, and cov(*ε_ijklmn_*,*ε_i’j’k’l’m’n’_*) = 0, otherwise:

logit(*y_ijklmn_*) = *µ* + *P_i_* + *D_j_*(*P_i_*) + *F_k_* + *G_l_* + *P_i_***G_l_* + *O_m_* + *ε_ijklmn_*,

where *y_ijklmn_* and *ε_ijklmn_* are respectively the *n*^th^ observation and corresponding model error at the *j*^th^ day inside the *i*^th^ period, in *l*^th^ group and *k*^th^ level of external disturbing factors fixed by *m*^th^ observer. The periods were compared applying the Tukey *post-hoc* test for pairwise comparisons on least square means.

The experimental unit in production data analyses was the cow with 12 test day milkings before and 12 after the transition, that is one year before and one year after the transition of the cows. The cows’ milk production and milk quality traits were analysed with the general linear mixed models considering fixed effects of parity *P_i_*, lactation month *LM_j_* and calendar month by calendar year by parity interaction *MY_k_***P_i_*, and non-zero covariance *σ*^2^*_C_* of model errors corresponding to recordings of the same cow, cov(*ε_ijkl_*,*ε_i’j’k’l’_*) = *σ*^2^*I(*i*=*i’* & *j*=*j’* & *k*=*k’* & *l*=*l’*) + *σ*^2^*_C_**I(*l*=*l’*), I() is indicator function:

*y_ijkl_* = *µ* + *P_i_* + *LM_j_* + *MY_k_***P_i_* + *ε_ijkl_*,

where *y_ijkl_* and *ε_ijkl_* are respectively the observation and corresponding model error of *l*^th^ cow from the *i*^th^ parity at the *j*^th^ lactation month and *k*^th^ calendar month by calendar year combination. The least square means for each calendar month and parity were estimated and the production traits measured at the last month before and at the first month after transition were compared with appropriately defined contrasts.

The experimental unit in cows’ health and body condition data analyses was the breed subgroup (two breeds) of observational group of cows who were observed repeatedly. Each subgroup was observed six times during period 1 and twice per period for periods 2–7. The lameness score was transformed into binary dummy variables and for each lameness score value the logistic model was fitted. All models considered effects of period *P_i_*, breed *B_j_* (the observations were made by breeds) and group *G_k_*, period by breed and period by group interaction effects *P_i_***B_j_* and *P_i_***G_k_*, and day nested to period effect *D_l_*(*P_i_*):

logit(*y_ijkl_*) = *µ* + *P_i_* + *B_j_* + *G_k_* + *P_i_***B_j_* + *P_i_***G_k_* + *D_l_*(*P_i_*) + *ε_ijkl_*.

The periods were compared applying the Tukey *post-hoc* test for pairwise comparisons on least square means.

The body condition score and cleanliness score were analysed with the general linear mixed models. The occurrences of skin lesions, leg lesions and udder problems were analysed with the generalized linear mixed models with logistic link function. All models considered fixed effects of month relative to transition *M_i_*, breed *B_j_* and month by breed interaction *M_i_***B_j_*, and random effect of observer *O_k_*~N(0,*σ*^2^*_O_*):

*y_ijkl_* = *µ* + *M_i_* + *B_j_* + *M_i_***B_j_* + *O_k_* + *ε_ijkl_*

and

logit(*y_ijkl_*) = *µ* + *M_i_* + *B_j_* + *M_i_***B_j_* + *O_k_* + *ε_ijkl_*,

where *y_ijkl_* and *ε_ijkl_* are respectively the *l*^th^ observation and corresponding model error of cow from the *j*^th^ breed at the *i*^th^ month observed by *k*^th^ observer. The months were compared applying the Tukey *post-hoc* test for pairwise comparisons on least square means.

All logistic models were fitted with SAS procedure GLIMMIX and all linear models with SAS procedure MIXED. Results were considered statistically significant at p≤0.05.
